# Curious Case of Strictured Œsophagus

**Published:** 1826-07

**Authors:** 


22. *Curious Case of Stricture of the Œsophagus.* M. De C. a Knight of St. Louis, was expatriated from his country in the revolution, and endured with fortitude the greatest hardships in a foreign land: yet he maintained excellent health till an advanced age, and in his 74th year travelled from Bourdeaux to Paris on foot. From his infancy, he had a difficulty of swallowing solid food, though this inconvenience appeared to produce no disorder of the digestive or other functions. In his 76th year, an attempt was made to supply him with a set of artificial teeth, which did not succeed, and produced inflammation which spread successively to all parts of the alimentary canal, and during several months he was a prey to vomiting and gastric derangement immediately on taking any food. These symptoms at length became mitigated; and he entered the MAISON DE SANTÉ under M. Dumeril, still preserving a pressing desire for food, but condemned to liquids. Even these he swallowed with difficulty, and with a kind of gurgling noise. Immediately after taking any substance into the pharyngeal cavity, it kept constantly regurgitating into the mouth, accompanied by a great flow of saliva. He was generally obliged to use frictions to the sides of the neck, to facilitate the deglutition. In this way he went on leading a miserable life, and repeatedly imploring that a bougie should be passed down the œsophagus, to try to remove the obstruction. But neither M. Dumeril nor M. Dubois, who was consulted, would make the attempt, a circumstance that surprises us very much. At length he died of the exhaustion.

*Dissection.* The lower portion of the pharynx presented a very considerable dilatation or pouch, and there was seen a stricture so narrow as scarcely to admit a probe. There was no change of structure in the parietes of the œsophagus constituting the stricture, which appeared to be a simple contraction, not dependent on any organic alteration of parts.

This is certainly a rare disease. Dr. Baillie, however, mentions this kind of stricture of the œsophagus, which he attributes to simple contraction of its muscular fibres. There are also a few others on record. We do not see any reason why the bougie should not have been tried in this case. Surely it might have been at least as serviceable as in an organic stricture.

